# Advances in the application of recombinase-aided amplification combined with CRISPR-Cas technology in quick detection of pathogenic microbes

**DOI:** 10.3389/fbioe.2023.1215466

**Published:** 2023-08-31

**Authors:** Xiaoping Li, Shuying Zhu, Xinling Zhang, Yanli Ren, Jing He, Jiawei Zhou, Liliang Yin, Gang Wang, Tian Zhong, Ling Wang, Ying Xiao, Chunying Zhu, Chengliang Yin, Xi Yu

**Affiliations:** ^1^ Faculty of Medicine, Macau University of Science and Technology, Avenida Wai Long Taipa, Macau, 999078, China; ^2^ Key Laboratory of Pollution Exposure and Health Intervention of Zhejiang Province, Shulan International Medical College, Zhejiang Shuren University, Hangzhou, Zhejiang Province, 310015, China; ^3^ State Key Laboratory for Diagnosis and Treatment of Infectious Diseases, National Clinical Research Center for Infectious Diseases, The First Affiliated Hospital, Zhejiang University School of Medicine, Hangzhou, Zhejiang Province, 310003, China; ^4^ Guangdong-Hong Kong-Macau Joint Laboratory for Contaminants Exposure and Health, Guangzhou, Guangdong Province, 510006, China; ^5^ Clinical Psychology Department, The Affiliated Hospital of Hangzhou Normal University, Hangzhou, Zhejiang Province, 310005, China

**Keywords:** recombinase-aided amplification, clustered regularly interspaced short palindromic repeats associated proteins, pathogenic microbes, quick detection, specific diagnosis

## Abstract

The rapid diagnosis of pathogenic infections plays a vital role in disease prevention, control, and public health safety. Recombinase-aided amplification (RAA) is an innovative isothermal nucleic acid amplification technology capable of fast DNA or RNA amplification at low temperatures. RAA offers advantages such as simplicity, speed, precision, energy efficiency, and convenient operation. This technology relies on four essential components: recombinase, single-stranded DNA-binding protein (SSB), DNA polymerase, and deoxyribonucleoside triphosphates, which collectively replace the laborious thermal cycling process of traditional polymerase chain reaction (PCR). In recent years, the CRISPR-Cas (clustered regularly interspaced short palindromic repeats-associated proteins) system, a groundbreaking genome engineering tool, has garnered widespread attention across biotechnology, agriculture, and medicine. Increasingly, researchers have integrated the recombinase polymerase amplification system (or RAA system) with CRISPR technology, enabling more convenient and intuitive determination of detection results. This integration has significantly expanded the application of RAA in pathogen detection. The step-by-step operation of these two systems has been successfully employed for molecular diagnosis of pathogenic microbes, while the single-tube one-step method holds promise for efficient pathogen detection. This paper provides a comprehensive review of RAA combined with CRISPR-Cas and its applications in pathogen detection, aiming to serve as a valuable reference for further research in related fields.

## 1 Introduction

With the continuous development of the Chinese social economy, animal husbandry has become more and more large-scale, intensive, and specialized, and the occurrence of animal epidemics has also increased, which has seriously hindered the sustainable and healthy development of Chinese animal husbandry. At the same time, most animal pathogens can also cause human diseases, such as *Brucella* (Abedi et al., 2020), rabies virus (Fisher et al., 2018), Toxoplasma gondii (Elsheikha et al., 2021), etc. People can become infected by touching infected animals or eating pathogen-containing meat. Therefore, establishing rapid and effective diagnostic methods is an important prerequisite to preventing and controlling the spread of pathogenic microbes.

Currently, microscopic detection technology and serological detection technology are two conventional pathogen detection technologies. However, the operation of microscopic inspection technology is complex and vulnerable to human factors. Although serological detection technology has the advantages of high sensitivity, rapidity, and intuition, it also has the disadvantage that infection cannot be detected at an early stage because antibodies need several days to produce (Lee et al., 2020a). With the rapid development of molecular biology technology, some new molecular biology detection technologies have emerged and have been widely used (Umesha and Manukumar, 2018). RAA is a new molecular biological detection technology. Compared to traditional PCR technology, it does not need expensive experimental equipment and consumables and has a wider application range and easier operation. At present, loop-mediated isothermal amplification (LAMP), recombinase polymerase amplification (RPA), and RAA are several mature isothermal nucleic acid detection technologies (Yan et al., 2014). They are isothermal, specific, and efficient, among which RAA is the only backup technology that is possible to carry out *in vivo* nucleic acid amplification technology (Gui et al., 2022).

RAA is a new isothermal nucleic acid rapid amplification technology. Rapid amplification of DNA or RNA can be achieved at a lower temperature (generally 37°C). The reaction speed is fast and the amplification product can be obtained in 30 min. This technology must use four core substances, namely recombinase, SSB, DNA polymerase, and deoxyribonucleoside triphosphate (dNTP). These four substances can replace the chain-breaking thermal cycle process of ordinary PCR. CRISPR-Cas as a system was originally found and whereafter was taken as a useful tool for genome editing and nucleic acid diagnosis owing to its reliability, sensitivity, and specificity. Multiple types of CRISPR-Cas systems including Cas9, Cas12a, Cas12b, Cas13, and Cas14 are successively developed for bacteria, viruses, and parasite detections. In addition, RAA can be combined with CRISPR-Cas and other new detection technologies to make pathogen detection more convenient and efficient, and its portable detection instrument also provides the possibility of on-site detection (Zhang et al., 2022a).

RAA (Recombinase Polymerase Amplification) plays a crucial role in amplifying the initial signals by increasing the concentration of the template for the Cas proteins. In diagnostics, two key players are Cas12a and Cas13a, which are known for their collateral cleavage activity (Kaminski et al., 2021; Karmakar et al., 2022). One of the notable advancements in Cas12a-based diagnostic systems is the DNA Endonuclease-Targeted CRISPR Trans Reporter (DETECTR). DETECTOR uses Cas12a, which recognizes the target dsDNA and indiscriminately cuts through nearby single-stranded DNA. In DETECTOR, Cas12a is guided to the target dsDNA by a complementary guide RNA. Once Cas12a binds to the correct target, it collateral cleaves ssDNA reporter molecules that are fused with a quencher and a fluorophore. The indiscriminate cleavage leads to the separation of the quencher from the fluorophore, which can be detected through the fluorescence signal. The sensitivity of DETECTR can be further enhanced by combining it with RPA/RAA preamplification. This technique has demonstrated successful detection of human papillomavirus and SARS-CoV-2 (Broughton et al., 2020).

Another powerful diagnostic tool harnessing collateral cleavage activity is the Specific High-sensitivity Enzymatic Reporter unLOCKing (SHERLOCK), which is based on the Cas13a protein (Gootenberg et al., 2017). Similar to Cas12a, Cas13a exhibits indiscriminate cleavage of nearby single-stranded RNA upon recognizing the target RNA. SHERLOCK has been used for the sensitive detection of disease-causing bacteria such as *M. tuberculosis* and *K. pneumoniae*, as well as viruses like SARS-CoV-2 (Xiang et al., 2020). The SHERLOCK method involves isothermal amplification of collected samples using RPA/RAA. Subsequently, an engineered Cas13a, a guide RNA, an RNA reporter, and a reporter probe containing a short oligonucleotide separating a fluorophore and a quencher are added to the sample. Once Cas13a binds to its specific target, it initiates the indiscriminate splicing of nearby RNA, including the reporter. This leads to the separation of the reporter and the quencher, generating distinct signals within the sample (Patchsung et al., 2020; An et al., 2021).

This review paper aims to provide an overview of the principle, characteristics, derivative technologies, and applications of RAA combined with CRISPR-Cas technology in the detection of pathogenic microbes. It seeks to serve as a valuable reference for related research in this field.

## 2 Exploration of the RAA technical principle and conditions

### 2.1 The replication mechanism

**FIGURE 1:**
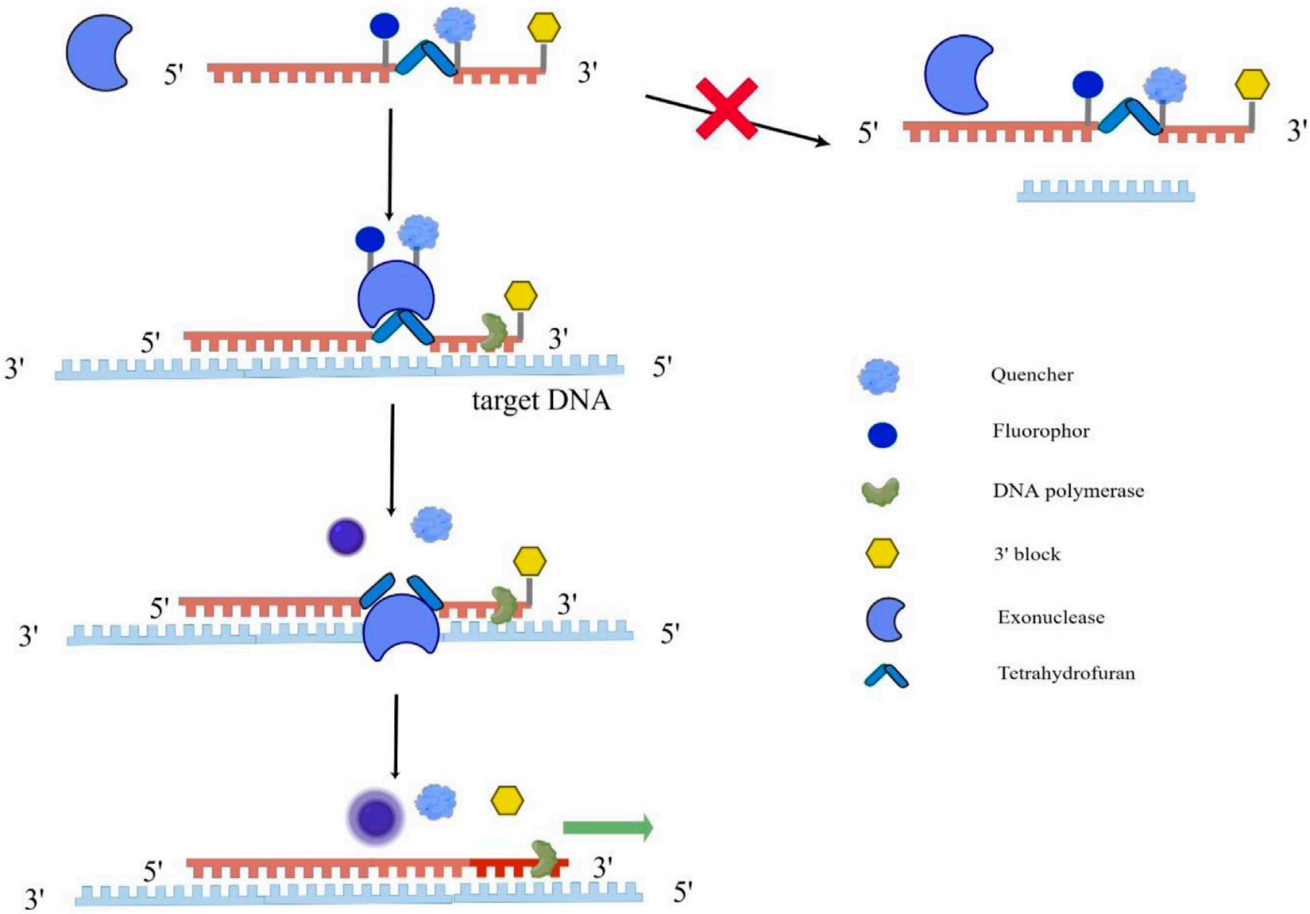
Schematic diagram of RAA implementation for real-time detection. This figure was drawn by Figdraw. The single fluorogenic probe includes fluorophor, quencher and 3′block, and exonuclease cannot recognize the single fluorogenic probe. As the probe binds to the target DNA, exonuclease recognizes tetrahydrofuran on the probe abasic site and cleavage them, allowing the fluorophor and quencher to separate and fluoresce. At the same time, DNA polymerase unblocks the 3′block and completes the chain extension.

RAA technology mainly uses recombinase, single-strand binding protein, and DNA polymerase to amplify the target gene. First, under an isothermal 37°C condition, the recombinase (from bacteria or fungi) and the primer DNA were closely combined to form a primer polymer; The next step is the template unwinding process. When the primer specifically recognizes the complementary sequence, the template DNA will unwind with the help of recombinase; Then, under the action of dNTP and energy, DNA polymerase completes the chain extension to form a new chain. The whole reaction is usually carried out at 37°C for 30 min, and a large number of amplification products can be obtained. From the working principle of RAA, it can be seen that once amplification conditions are fulfilled, the reaction products will increase exponentially. But how can RAA achieve real-time detection at this time? The real-time detection incorporates a fluorogenic probe besides forward and reverse primers, and the reaction initiates based on the cleavage of probe at an abasic site (Tetrahydrofuran, THF) between the fluorophor and the quencher with exonuclease (Figure 1) (Fan et al., 2020; Mao et al., 2022).

### 2.2 The design of RAA primers

Certain principles should be followed when designing the RAA primer, and it is different from the design of conventional PCR primers. First, the RAA primer’s length is generally 30–35 nt. Too short of a primer will affect the combination of primer and recombinase. Too long primers are easy to form primer dimers, leading to an increase in false positive rate. In addition, when designing RAA primers, it is better not to have a palindrome sequence, a continuous single-base repeat sequence, and an internal secondary structure region in the sequence, and the melting temperature (Tm value) is not an important factor to be considered. Because the amplification efficiency of different primers is different, it is usually necessary to design a series of candidate primers for an amplified gene, and finally select the best primer pair through experimental optimization (Xia et al., 2022).

### 2.3 The template for the RAA reaction

In the RAA reaction system, the length of the target gene is generally 100 to 200 bp; to ensure rapid and effective amplification of the template for RAA, the maximum length should not exceed 500 bp, because the long target fragment will reduce the rate of the whole reaction.

## 3 Advantages and disadvantages of RAA technology

The RAA assay, as a novel isothermal amplification technology, has been widely employed for detecting various pathogens. Unlike the RPA assay, the RAA assay utilizes three core enzymes: recombinase UvsX, DNA polymerase, and SSB (Xue et al., 2020a). Wang et al. (2020) successfully developed an RAA assay for the detection of Orf virus (ORFV). This assay demonstrates a rapid detection time of 30 min and a detection limit of 10 copies per reaction. Importantly, it exhibits no cross-reactivity with other common DNA viruses. Consequently, the RAA assay proves to be a fast, sensitive, and specific approach for clinical testing of ORFV. In comparison to LAMP, RAA is simpler to conduct, requiring only a pair of primers, operating at a lower temperature range of 37°C–42°C, and yielding shorter run times of less than 30 min. In contrast, LAMP necessitates four or six primers, operates at a higher temperature of 65°C, and has a longer run time (Pang and Long, 2023). The advantage of RAA technology is that the reaction can be amplified at the optimized 37°C or room temperature, the target amplification product can be obtained in 30 min, and the product grows exponentially without any auxiliary heating equipment, which is particularly suitable for on-site detection of pathogens. Furthermore, the primers in the RAA reaction system must be strictly complementary to the template, and the length is 30–35 nt, which ensures the precision and specificity of the RAA method.

However, because the sensitivity of RAA is very high, it is easy to cause false positive results, so cross-contamination should be avoided throughout the process. Another disadvantage of RAA, it is easy to produce nonspecific products in the reaction process, which is mainly due to the short length of the target gene (Jaroenram and Owens, 2014). In addition, the formation of primer dimers is caused by excessive primers and specific interactions between primer molecules (Kellner et al., 2019). Currently, there are significant challenges associated with RAA. Firstly, achieving on-site sample pre-treatment, such as nucleic acid extraction, is difficult. This step is crucial to obtain high-quality nucleic acid templates for amplification. Overcoming this challenge is essential for enabling the use of RAA in various settings where immediate sample processing is required. Secondly, an urgent technical challenge that needs to be addressed is how to achieve multi-target isothermal amplification detection under single-tube closed-tube conditions with high sensitivity and specificity. The ability to detect multiple targets simultaneously within a single reaction is highly desirable for efficient and comprehensive pathogen identification. Without a solution to this challenge, the application range of RAA may be limited. Addressing these issues is critical to expanding the application range of RAA and maximizing its potential as a powerful diagnostic tool. Ongoing research and development efforts are focused on overcoming these limitations to further enhance the utility and effectiveness of RAA in practical settings.

## 4 Derivative technologies based on RAA technology

The target gene product of traditional RAA technology is mainly judged by gel electrophoresis, but with the rapid development of this technology, traditional RAA technology can be combined with other new technologies, including the real-time fluorescent RAA, RT-RAA, RAA auxiliary CRISPR-Cas13a/Cas12a method, which determines detection results more convenient and intuitive. At the same time, it has greatly expanded the application of this technology in pathogen detection.

### 4.1 Real-time fluorescent RAA

Real-time fluorescent RAA technology is a combination of the basic reaction system of RAA and the fluorescent probe system, which can monitor the amplification results of target gene fragments in real-time and fully meet the needs of on-site detection. In 2022, Wang et al. (2022b) used real-time fluorescent RAA to rapidly detect Senecavirus A. The sensitivity of this method is the same as that of the real-time fluorescent quantitative PCR method, and there is no cross-reaction with other types of respiratory viruses. Furthermore, RAA technology can also be used together with a fluorometer to mark different target genes with different fluorescence colors in the same reaction, which can form a set of RAA multiple fluorescence real-time monitoring systems, while other isothermal nucleic acid amplification technology or Nest PCR technology cannot achieve this effect (Seo et al., 2022).

### 4.2 RT-RAA

Reverse transcriptase recombinase-aided amplification (RT-RAA) technology is based on RAA technology. Its principle is that first the pathogen’s RNA is reverse transcribed into cDNA, and then recombinase was used to replace the high-temperature denatured quantitative PCR (qPCR) to complete the template chain breaking process. Then, under the action of the SSB, the DNA polymerase completes the chain extension, and finally, the new chain can be generated. Chen et al. (2018a) used RT-RAA technology to rapidly detect different subgroups of respiratory syncytial virus (RSV) and the results were consistent with those of real-time qPCR (RT-qPCR). In addition, this technology can also be combined with lateral flow immunoassay (LFIA) technology to make the determination of the result more intuitive. In 2020, Xiong et al. (2020) successfully established a rapid and intuitive detection method for dengue virus (DENV) based on RT-RAA and LFIA. This method only needs to react at 37°C for 20 min, then it can be visually observed by LFD in 3 min. It has the advantages of strong specificity and high sensitivity and is especially suitable for the accurate detection of DENV when laboratory resources are scarce.

### 4.3 RAA combined with CRISPR-Cas13a/Cas12a method (RAA-Cas13a/Cas12a)

In recent years, clustered regularly interspaced short palindromic repeats associated proteins (CRISPR-Cas), as a genome engineering tool that revolutionizes life science, has received extensive attention in the fields of biotechnology, agriculture, and medicine (Hille et al., 2018). CRISPR-associated protein 13a (Cas13a or C2c2) is a class VI-A protein, which can be used not only for gene editing but also for the detection of pathogenic nucleic acids (Gootenberg et al., 2017; Myhrvold et al., 2018). CRISPR-associated protein 12a (Cas12a or Cpf1) is a kind of RNA-guided enzyme that can specifically bind and cut target DNA and is an important part of the adaptive bacterial immune system. Cas12a protein, known as Cpf1, is widely utilized in genome editing due to its ability to trigger double-stranded DNA breakage at specific locations (Chen et al., 2018a). Bai et al. (2019) combined RAA technology with CRISPR-Cas to establish a diagnosis system of RAA-Cas12a, which can quickly and easily detect the African swine fever virus. Furthermore, Chen et al. (2022) successfully established a quick, sensitive, and specific detection method for SARS-CoV-2 using RAA technology and the CRISPR-Cas detection system. The sensitivity of the RAA-Cas13a method can reach 10 copies of RNA molecules/reaction (Li et al., 2022). It summarizes all important reports about the application of RAA combined with CRISPR-Cas12a/Cas13a in the quick detection of pathogenic microbes (Table 1) (Li et al., 2021; Wang et al., 2022c; Zhang et al., 2022b; Chen et al., 2022; Fang et al., 2022; Qian et al., 2022; Wei et al., 2022; Xing et al., 2023; Zhao et al., 2023).

**TABLE 1:** The application of RAA combined with CRISPR-Cas12a/13a technology in the quick detection of pathogenic microbes.

RAA- Cas12a/Cas13a	Signal approach	Pathogens	Limit of detection (copies/mL)	Time (min)	References
RAA-Cas12a	Fluorescence	*Norovirus*	1.0×10^2^	40	Qian et al. (2022)
RAA-Cas12a	Fluorescence	*Methicillin-resistant Staphylococcus aureus*	1.0×10^4^	60	Wang et al. (2022b)
RAA-Cas12a	finger-actuated microfluidic biosensor (FA-MB)	*Bacillus cereus, Pseudomonas aeruginosa, Salmonella typhimurium,* et al.	2.5 × 105	60	Xing et al. (2023)
RAA-Cas12a	Electrochemical biosensor (E-CRISPR)	*L. monocytogenes*	1.3×10^4^	45	Li et al. (2021)
RAA-Cas12a	Centrifugal microfluidics	*SARS-CoV-2*	1.0×10^3^	30	Chen et al. (2022)
RAA-Cas12a	Fluorescence	*Escherichia coli O157:H7*	2.7×10^5^	30	Fang et al. (2022)
RAA-Cas12a	Fluorescence	*Monkeypox virus*	1.0×10^3^	75	Zhao et al. (2023)
RAA-Cas13a	Fluorescence	*Encephalomyocarditis virus*	1.0×10^3^	60	Wei et al. (2022)
RAA-Cas13a	fluorescent visualizers	*SARS-CoV-2*	1.0×10^4^	60	Zhang et al. (2022b)

## 5 Application of RAA technology

Since the advent of RAA technology, it has been widely used in the detection of pathogenic microbes, including bacteria (Zhang et al., 2017), viruses (Shen et al., 2019), parasites (Lin et al., 2022), and other pathogens. Furthermore, this technology can also be applied to SNP and cancer detection measure (Duan et al., 2018).

### 5.1 Bacteria detection

*Salmonella* is a kind of non-spore Gram-negative *bacillus*, distributed throughout the world and mainly parasitizes human and animal intestines, posing a serious threat to human and animal health (Yin and Zhou, 2018). *Salmonella* laboratory diagnosis methods rely mainly on microbial culture and serological detection technology from blood or bone marrow (Crump et al., 2015). With the continuous development of molecular biological technology, Zhang et al. (2017) successfully established a molecular biological method for the rapid detection of *salmonella* by RAA. The whole reaction can completely amplify a large number of target genes within 20 min, and it has high sensitivity and good specificity. The lower limit for *salmonella* detection is 102 copies/μL. There was no cross-reaction with *Escherichia coli* and *Shigella*. This method provides powerful technical support for the molecular diagnosis of *Salmonella*.

### 5.2 Virus detection

Severe acute respiratory syndrome coronavirus 2 (SARS-CoV-2) was first discovered in Wuhan, China. The 99 patients admitted to Wuhan Jinyintan Hospital were the first confirmed patients reported with Corona Virus Disease 2019 (COVID-19) (Chen et al., 2020). The harm of SARS-CoV-2 manifests itself primarily in systemic infection of many animals and human respiratory tract infections, such as severe acute respiratory syndrome (SARS) and Middle East respiratory syndrome (MERS) (Drosten et al., 2003; Zaki et al., 2012; Yin and Wunderink, 2018). Clinical manifestations of COVID-19 are flu-like symptoms such as fever, cough, fatigue, dyspnea, and gastrointestinal symptoms (Huang et al., 2020). In 2022, Yao et al. (2022) designed two sets of RT-RAA primers and probes based on the ORF1ab gene and the N gene, and successfully established a rapid RT-RAA detection method for SARS-CoV-2. This method is specific and sensitive. It has no cross-reaction with other respiratory pathogens and has the same sensitivity as RT-qPCR. This study provides a simple and reliable method for the detection of SARS-CoV-2. This method is suitable for diagnostic laboratories with insufficient equipment and provides technical support for clinical treatment and disease control in grass-roots hospitals in the future (Li et al., 2022). The avian influenza virus (AIV) is an RNA virus that belongs to the influenza A virus causing zoonotic diseases. In the past 20 years, avian influenza has been ravaging the world, and new AIV has also been discovered (Yang et al., 2023), becoming a global public health problem and attracting high attention from the international community (Domanska-Blicharz et al., 2023; Tahmo et al., 2023). Wang et al. (2022a) established a sensitive and specific detection method for H5 subtype AIV with the RT-RAA method for the first time. The results showed that H5 subtype AIV detection by the RT-RAA method could produce positive signals in 20 min, and the whole reaction could be completed in about 30 min. While RT-qPCR takes at least 2 h. Furthermore, the cost of RT-RAA detection is almost half that of RT-qPCR. This is a comparison of clinical applications between the RT-RAA method and the RT-qPCR method (Table 2).

**TABLE 2:** Comparison of clinical applications between RT-RAA and RT-qPCR.

Compare items	RT-RAA	RT-qPCR	References
Principle differences	SSB keeps the template in a single-stranded notch state and nucleic acid amplification is uninterrupted at 37°C.	Reamplification required chain denaturation at 94°C.	Zhang et al. (2017)
Reaction process	In the whole reaction process, RNA can be directly used as a template to be added to the amplification system, and reverse transcription and amplification are carried out simultaneously.	For RNA amplification, it is necessary to perform reverse transcription reaction first, which is to reverse transcribe RNA into cDNA, and then use cDNA as template for nucleic acid amplification.	Li et al. (2022)
Reaction temperature	The reaction can be carried out at a constant temperature ranging from 37°C to 42°C.	Three temperatures are required for continuous circulation, such as 95°C, 72°C, 55°C, and the temperature must be precisely controlled.	Shen et al. (2019)
Reaction time	20 min.	At least 120 min.	Chen et al. (2020)
Detection equipment	Small fluorescent detector.	RT-qPCR instrument.	Yao et al. (2022)
Detection site	On site.	PCR special laboratory.	Tang et al. (2021)
Detection personnel	On-site personnel.	Medical professional.	Lee et al. (2020b)

### 5.3 Parasite detection

Malaria is a serious global vector disease caused by plasmodium. Plasmodium is a kind of single-celled protozoa, mainly distributed in tropical and subtropical regions, covering more than 90 countries and regions around the world, with 224 million infected people, most of whom are children (Lee et al., 2020b; Naserrudin et al., 2022). The early detection of malaria parasites is mainly by microscopy, but this method is time-consuming, laborious, and subject to human factors. Since the advent of RAA technology, this method has been widely used in the pathogen detection (Allen et al., 2022). In 2022, Lin et al. (2022) used the search in the NCBI database to obtain multiple genomic sequences of 18SrDNA from malaria parasites, designed universal primers to detect malaria parasites, and successfully established a detection method for malaria parasites using RAA. It can be seen that this technology provides a powerful technical means for effective prevention and control of parasites.

## 6 Discussion

**FIGURE 2:**
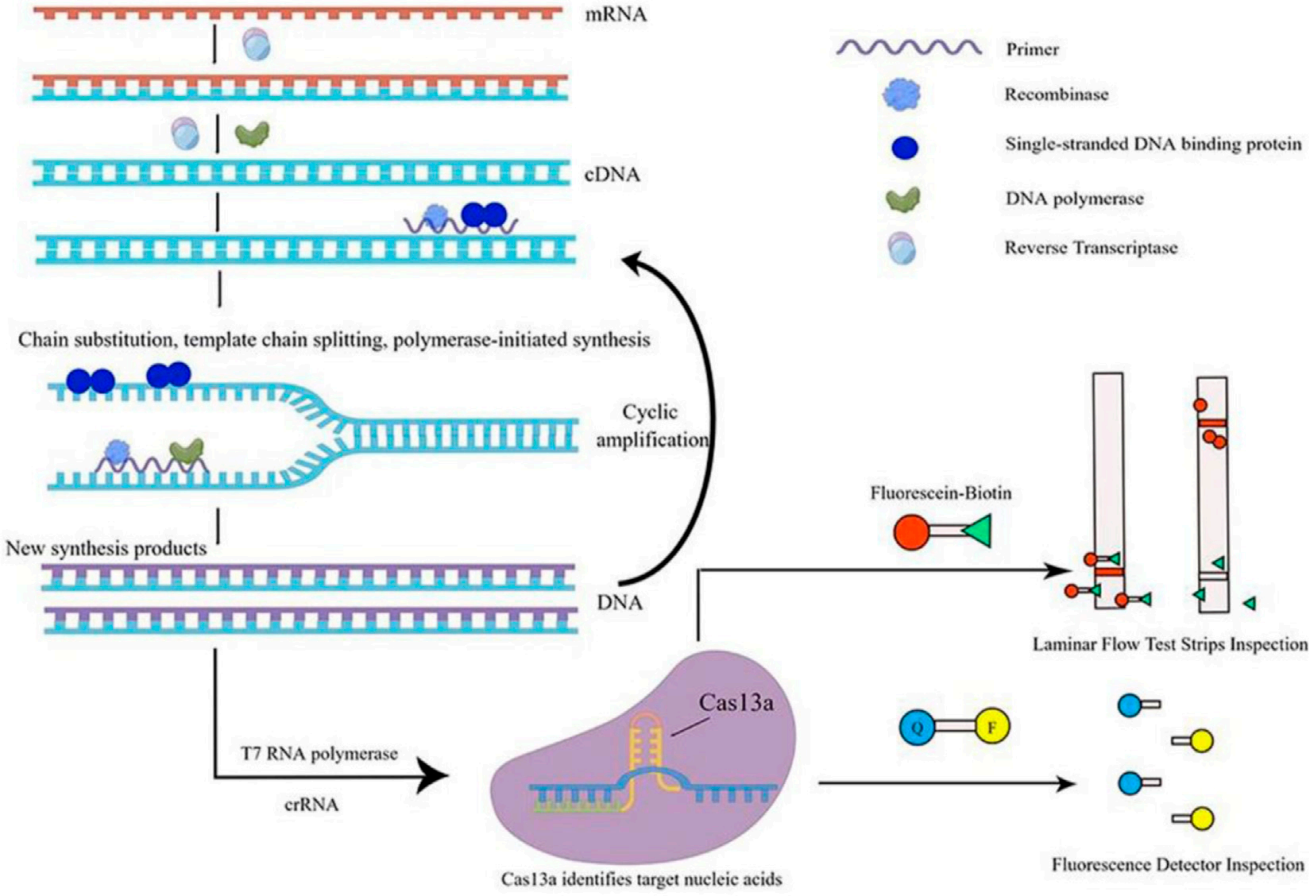
Schematic diagram of single-tube one-step method. This figure was drawn by Figdraw. The specific principle of single-tube one-step detection of RNA viruses is as follows: in the nucleic acid amplification system under constant temperature conditions, firstly, dNTP is used as substrate material, mRNA is used as a template, and under the action of reverse transcriptase, a cDNA single-strand complementary to the RNA template is synthesized to form DNA-RNA heterozygote. In this DNA-RNA heterozygote, RNA is specifically degraded by reverse transcriptase, and then dNTP is used as the substrate, the first strand of cDNA is used as a template, and under the action of DNA polymerase, the second strand of cDNA is synthesized, and finally the double-stranded DNA molecule is formed, that is, the DNA synthesis process guided by RNA is completed. Then, this double-stranded DNA molecule is used as the template DNA. When the primer finds the complementary sequence that perfectly matches the template DNA, with the help of recombinase, the double-stranded structure of the template DNA is opened, while SSB stabilizes the displaced DNA chain, and the extension of the strand is completed under the action of DNA polymerase to form a new complementary strand of DNA. The T7 RNA polymerase transcribed the amplified product into RNA, while the Cas13a protein specifically bound the target nucleic acid under the guidance of crRNA, the protein structure was changed, and was converted into ribonuclease, which was non-specific to cut RNA. The incidental cutting activity could cut the introduced externally derived FQ-coated ssRNA probe. The fluorescence signal can be amplified rapidly to detect the target nucleic acid quickly.

In recent years, there has been an increasing number of studies that have integrated the RPA or RAA system with the CRISPR-Cas system. Cas13a protein, renowned for its collateral cleavage activity, serves as a key component in diagnostics. RPA/RAA aids in amplifying the initial signals by enhancing the template concentration for Cas13a. The sequential implementation of these two systems is employed for the molecular diagnosis of pathogenic microbes (Kirchner and Schneider, 2015). Among them, RAA technology uses reverse transcriptase (RT), recombinase, single-stranded DNA binding proteins (SSB), DNA polymerase, etc. It can amplify the target gene by millions or even tens of millions of times in 30 min under isothermal conditions (37°C). Thus, the mixed system ‘RPA/RAA and CRISPR-Cas13a′ can achieve the goal of high specificity and quick detection (Gootenberg et al., 2017). However, at present, the detection of pathogenic microbes only achieves the step-by-step operation of RAA and CRISPR-Cas13a: first, the RAA system is used to rapidly isothermal amplify the nucleic acid of pathogenic microbes, and then the CRISPR-Cas13a system is used to quickly identify and detect products amplified by RAA. The nonintegrated detection method has been applied to the quantitative detection of adenovirus, *Staphylococcus aureus*, *Shigella*, and *Vibrio cholerae* (Xue et al., 2020b; Fan et al., 2021; Tang et al., 2021). It is precise because of the two-step operation of RAA and CRISPR-Cas13a (after the nucleic acid of pathogenic microbes has been amplified by RAA, it is necessary to open the cap and transfer the solution to the CRISPR-Cas13a tube for detection), which is prone to pollution. It is necessary to optimize the performance parameters of the two mixed systems of RAA and CRISPR-Cas13a, which can integrate the two tubes into one tube (single tube). This single-tube one-step method is similar to the one-pot SHERLOCK of Zhang Feng’s team (Figure 2), which can implement the universal detection of pathogenic microbes at various points (Joung et al., 2020).

## 7 Summary and outlook

Pathogenic microbes in nature are varied and mutate rapidly, which is one of the main factors causing food and public health safety problems. In recent years, the infection of viruses, bacteria, and parasites not only seriously threatens human health, but also hinders the development of the Chinese aquaculture industry. Therefore, there is a need to develop a more quick and accurate method to detect pathogenic zoonotic microbes (Rees et al., 2021). Traditional pathogen detection relies mainly on microscopic detection technology, but this technology has high requirements for operators. With the continuous development of molecular biology, some new molecular biological technologies are emerging. Among them, RAA technology is a relatively mature isothermal amplification technology, which has the advantages of simple operation, high sensitivity, strong specificity, and quick detection and can be used for on-site detection (Dinnes et al., 2022). Therefore, this technology is widely used to quickly detect viruses, bacteria, and parasites. With the continuous development and improvement of RAA technology, especially the improvement of the purity of some enzymes and proteins in the two systems (RAA and CRISPR-Casl3a) and the optimization of some conditions and parameters of the mixed system, it could be possible to establish a single-tube one-step method for quick detection of various viruses. This innovative single-tube one-step method technology and its derivative technology will have broad application prospects in the detection of pathogenic microbes (Gootenberg et al., 2018; Tao et al., 2020).
